# Deep learning-assisted object recognition with hybrid triboelectric-capacitive tactile sensor

**DOI:** 10.1038/s41378-024-00813-2

**Published:** 2024-11-07

**Authors:** Yating Xie, Hongyu Cheng, Chaocheng Yuan, Limin Zheng, Zhengchun Peng, Bo Meng

**Affiliations:** Key Laboratory of Optoelectronic Devices and Systems of Ministry of Education and Guangdong Province, College of Physics and Optoelectronic Engineering, Shenzhen University, Shenzhen, 518060 China

**Keywords:** Nanoscience and technology, Engineering

## Abstract

Tactile sensors play a critical role in robotic intelligence and human-machine interaction. In this manuscript, we propose a hybrid tactile sensor by integrating a triboelectric sensing unit and a capacitive sensing unit based on porous PDMS. The triboelectric sensing unit is sensitive to the surface material and texture of the grasped objects, while the capacitive sensing unit responds to the object’s hardness. By combining signals from the two sensing units, tactile object recognition can be achieved among not only different objects but also the same object in different states. In addition, both the triboelectric layer and the capacitor dielectric layer were fabricated through the same manufacturing process. Furthermore, deep learning was employed to assist the tactile sensor in accurate object recognition. As a demonstration, the identification of 12 samples was implemented using this hybrid tactile sensor, and an recognition accuracy of 98.46% was achieved. Overall, the proposed hybrid tactile sensor has shown great potential in robotic perception and tactile intelligence.

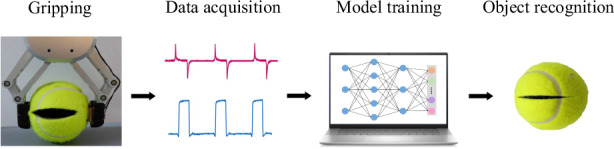

## Introduction

The rocketing development of human-machine interaction necessitates a great number of sensors for comprehensive information acquisition. There are mainly auditory, visual, and tactile sensors. As an important application for human-machine interaction and robotic intelligence, the development of object recognition technology can provide more intelligent interaction for intelligent robots and systems^1,2^. Tactile sensors can convert mechanical stimuli into electrical signals that can be used to detect information in object interactions, such as temperature^3–5^, humidity^6,7^, shape^8,9^, surface texture^10–12^ and softness^13,14^. This characteristic makes tactile sensing technology play an increasingly important role in the field of object recognition.

Various tactile sensors can be categorized into piezoresistive^15–17^, piezoelectric^18–20^, capacitive^21,22^, and triboelectric sensors^23–30^ based on their mechanisms. They can accomplish preliminary tactile perception and object recognition. Previous studies have investigated the utilization of tactile sensors for object recognition. Tactile sensors usually distinguish between different objects by some physical properties inherent in the objects^24^. Recognizing the shape of an object by using its shape features is a common method^8,31–33^. The surface texture is also used as a feature to obtain information about the contacted objects by sliding and pressing^34,35^. In addition, the hardness of the object is also one of the important features in object recognition^36–38^. These features are interrelated and form an overall characterization of the object.

However, single-function sensors may struggle in complex environments with diverse objects, necessitating the consideration of multiple parameters for accurate recognition. Therefore, in the process of object recognition, the comprehensive consideration of multiple aspects of the characteristics can improve the accuracy of object recognition. In general, there are two ways to obtain multidimensional information about the object being contacted. The first involves constructing a multi-sensor system, integrating multiple independent sensors to comprehensively determine object characteristics^39,40^. While providing comprehensive information, this approach entails low integration and high complexity. Alternatively, designing hybrid tactile sensors capable of simultaneously acquiring multiple signals offers a streamlined solution. Hybrid tactile sensors offer ease of integration and provide a high degree of multidimensional information for object recognition. Lee’s group proposed a biomimetic piezoelectric tactile sensor that can be used to recognize features on the surface of an object. The roughness of the object's surface texture is then recognized by machine learning^41^. In addition to being an efficient energy harvesting technology^42,43^, triboelectrification also shows great potential for haptic sensing owing to its excellent dynamic response characteristics^30^. Ding’s group proposed a hybrid triboelectric and piezoresistive sensor and built a real-time sensing system that was implemented on a robot manipulator. It can complete the task of texture and materials recognition using a parallel residual convolutional neural network (PR-CNN)^44^. In our previous work^24^, we developed a triboelectric-inductive hybrid tactile sensor. This sensor can achieve object recognition task among several kinds of fruits with different packages with the assistance of machine learning.

Admittedly, current efforts in the field enable the simultaneous acquisition of multidimensional information about an object. However, solutions for obtaining various types of signals with a single sensor are relatively scarce. To address this, we propose a hybrid tactile sensor capable of accurately capturing diverse information about an object by integrating different sensing signals within a unified framework. It combines a triboelectric sensing unit with a capacitive sensing unit based on a porous PDMS preparation procedure. Triboelectric sensing signals exhibit sensitivity to surface material and texture. Meanwhile, the capacitive sensing unit measures the object’s hardness when subjected to mechanical stimuli from a robotic gripper. Subsequently, the deep learning method was employed to enhance the sensor’s performance and achieve accurate recognition of multiple samples.

## Results and discussion

### Design and fabrication of the hybrid tactile sensor

**Fig. 1:**
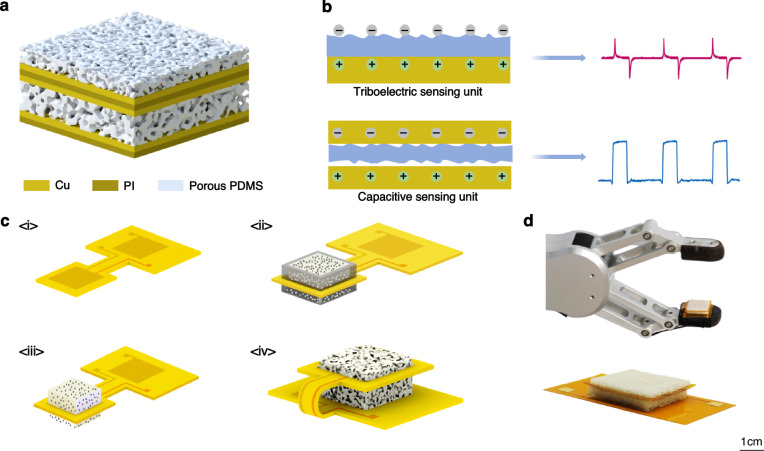
Design of the hybrid tactile sensor and the scheme of the process. **a** The structure of the tactile sensor. **b** Principle of capacitive sensing unit. **c** The fabrication process of hybrid tactile sensor. **d** Photographs of a fabricated hybrid tactile sensor

Figure 1a illustrates the schematic of the hybrid tactile sensor. It consists of a triboelectric sensing unit and a capacitive sensing unit. Such a combination does not require the introduction of functional materials. The friction layer of the triboelectric sensing unit and the dielectric layer of the capacitive sensing unit can be prepared using the same material and the same process, which greatly simplifies the preparation process of the hybrid sensor. Both the two sensing units are made of a porous PDMS sensitive film and copper electrodes. The triboelectric sensing unit is located on the upper layer, while the capacitive sensing unit is on the lower layer. The two sensing units are separated by a PI layer, which helps to avoid signal interference between the two sensing units. Figure 1b presents the operating principle of the triboelectric sensing unit and capacitive sensing unit. For the triboelectric unit, charges are transferred between the contacted surfaces when the sensor contacts with the object and separates. Differences in the surfaces’ ability to gain or lose electrons, as well as differences in the contact area caused by the object texture, can both lead to differences in triboelectric signals. Therefore, the triboelectric signal contains information about both the surface material and texture. Regarding the working mechanism of the capacitive unit, the porous PDMS is compressed when gripping objects and causes a reduction in the gap between the two electrodes. Consequently, this induces a change in its capacitance. When the same force is applied to objects with different hardness, the porous PDMS is compressed to different extents. Therefore, by observing changes in capacitance, the hardness of an object can be inferred. Such design guarantees that one mechanical stimulus converts into two kinds of signals. The bi-channel signals form a dual judgment for object recognition. This bi-channel signal fusion discrimination method provides a more comprehensive assessment of object properties, thereby enhancing the sensor’s applicability to a variety of objects.

Figure 1c presents a schematic of the sensor’s fabrication process based on a salting-out molding method. The fabrication started from a double-sided flexible copper clad laminate (FCCL) (Fig. 1c<i>). It was embedded in the upper and lower molds (Fig. 1c<ii>). After the PDMS mixture was poured into the molds and got cured, the molds were removed (Fig. 1c<iii>). After a salting-out process, porous PDMS films were fabricated. And, the FCCL on the other side was finally folded to obtain a complete hybrid tactile sensor (Fig. 1c<iv>). Figure 1d show photographs of a fabricated hybrid tactile sensor. The actual measured dimensions of the hybrid tactile sensor are shown in Fig. S1.

### Characterization of the tactile sensor

**Fig. 2:**
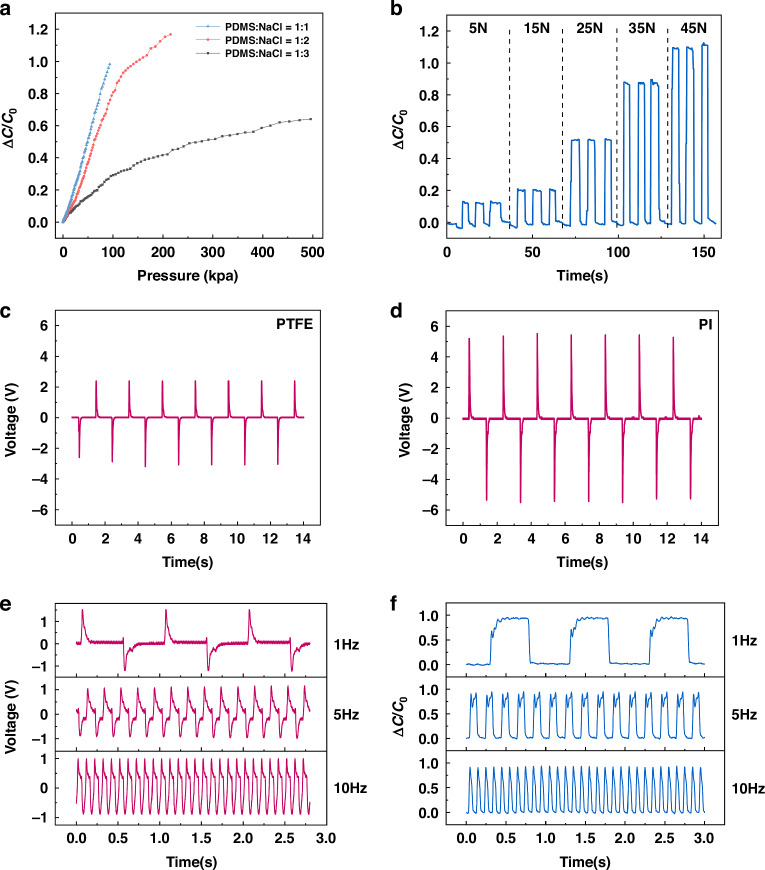
Characterization of the hybrid tactile sensor. **a** Capacitance variations of the capacitive sensing unit with different PDMS-to-NaCl mass ratios. **b** Capacitive variations of the capacitive sensing unit under progressively increasing pressures. **c**, **d** Output voltages of the triboelectric sensing unit in contact with PTFE and PI films at 0.5 Hz frequency. **e**, **f** Performance of the triboelectric sensing unit and capacitive sensing unit at various frequencies

First, we investigated the optimized mass ratio of PDMS-to-NaCl particles. Figure 2a illustrates the change of capacitance when the sensor with various PDMS-to-NaCl mass ratios under increasing applied pressures. It is apparent that the performance of hybrid tactile sensor varies with the mass ratios. The sensitivity increases with the PDMS-to-NaCl particle ratios. However, the measurement range shrinks as the ratios increase. The PDMS-to-NaCl particle ratios of 1:2 keep relatively high sensitivity in a larger measurement range. Thus, the same mass ratio was applied to the triboelectric sensing unit and all subsequent experimental data were obtained under this mass ratio.

An indenter with a contact area of 15 mm by 15 mm was employed to exert pressure on the hybrid tactile sensor. The compression force was systematically increased from 5 N to 45 N. As shown in Fig. 2b, the output signal of the capacitive sensing unit steadily increases with the rising pressure, highlighting the consistency and repeatability of the sensor.

The triboelectric sensing unit generates distinctive signals when in contact with objects of varying surface materials. Taking PI and PTFE as examples, the output voltage of the hybrid tactile sensor, when operated at a frequency of 0.5 Hz, is illustrated in Fig. 2c, d. When in contact with PTFE, the triboelectric sensing unit generates negative voltage, while positive voltage is produced upon separation. Conversely, the scenario is entirely reversed when in contact with and separating from PI. Significant differences are also observed in the peak-to-peak voltage between the two cases. This variation in output signal also appears on other surface materials. To confirm this sensitivity across various situations, we conducted experiments using different materials to touch the sensor’s surface periodically at a frequency of 0.5 Hz, as shown in Fig. S2. This validates that the sensor can detect these differences effectively. Fatigue tests of the sensor were carried out by involving 3000 cycles at a frequency of 0.5 Hz. As shown in Fig. S3, the bi-channel signals of the hybrid sensor demonstrate good resilience and robustness in operational conditions.

We also conducted tests on the characteristics of the hybrid sensor under various frequencies at fixed applied forces. The hybrid tactile sensor was mounted on a tensile testing platform, and PMMA was selected as the contact material. Figure 2e, f illustrates the responses obtained at frequencies of 1 Hz, 5 Hz, and 10 Hz, respectively. At these frequencies, both the two sensing units can generate stable signals. The triboelectric sensing unit demonstrates a more instantaneous and focused response, capturing the moment of contact and separation. Nevertheless, the capacitive sensing unit demonstrates a slower response speed, reflecting the feedback information of the object to applied forces. Such continuous response can thus provide information about the object’s hardness.

### Deep learning-assisted object recognition applications

In order to comprehensively evaluate the performance of the hybrid tactile sensors in object recognition, we built a platform as it’s shown in Figure S4. The hybrid tactile sensor was assembled on a robotic grip. Signal acquisition is facilitated through an oscilloscope and an impedance analyzer. This setup enables real-time capturing of triboelectric and capacitive signals, which is essential for accurate tactile sensing and object recognition tasks. In this platform, objects of varying hardness levels are tested, allowing for accurate comparative analysis in both tactile sensing and object recognition experiments.

**Fig. 3:**
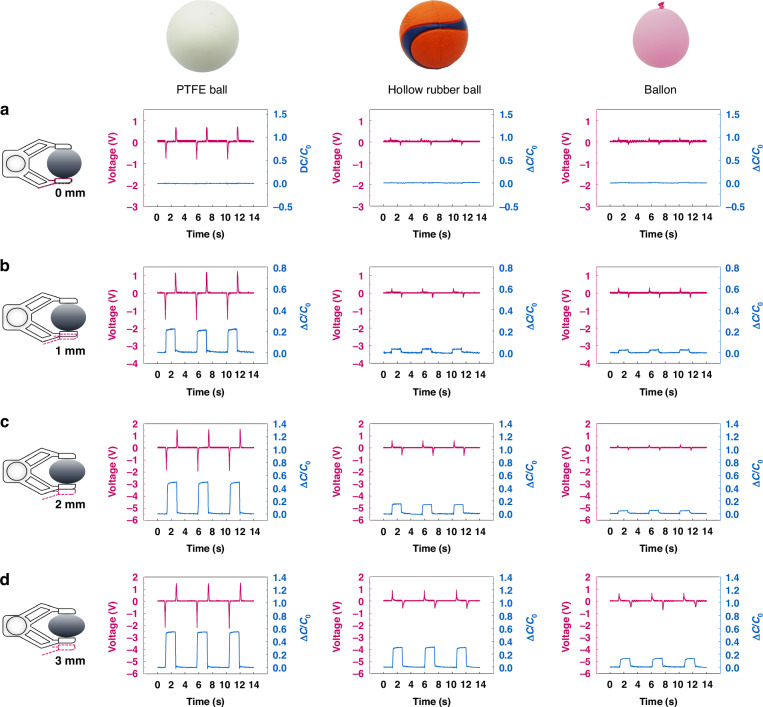
Optimization of the gripper's gripping depth. Bi-channel outputs of the sensor when gripping depth of the gripper is set as **a** 0 mm, **b** 1 mm, **c** 2 mm, and **d** 3 mm

To investigate the optimal gripping depths of the hybrid tactile sensor, we selected three samples with varying Shore hardness levels, including a PTFE ball, a hollow rubber ball, and a balloon. At an initial gripping depth of 0 mm, the sensor delicately brushes against the object’s surface. The triboelectric sensing unit generates a weak voltage signal, with negligible capacitance changes being observed (Fig. 3a). Upon increasing pressure to achieve a 1 mm gripping depth, the sensor enables nuanced detection of surface textures and material properties. Notably, gripping the PTFE ball elicits noticeable capacitance changes, while weaker signals are observed when gripping the hollow rubber ball and balloon. At this depth, the bi-channel signals of triboelectric and capacitance fail to distinguish among objects of different hardness (Fig. 3b). Further, augmenting pressure to attain a 2 mm gripping depth yields optimal sensitivity and discrimination of object compliance and deformability. The hybrid tactile sensor demonstrates stable grasping of objects with varying hardness levels, showcasing significant differences in bi-channel signals, particularly in distinguishing among objects of different Shore hardness (Fig. 3c). In contrast, increased pressure resulting in a 3 mm gripping depth compromises sensor performance, manifesting as reduced sensitivity and accuracy in tactile perception. Particularly for objects with higher Shore hardness, the hybrid tactile sensor exhibits diminished trends in capacitance and triboelectric signal changes compared to the 2 mm gripping depth (Fig. 3d). In the subsequent experiments, we chose 2 mm as the gripping depth to ensure reliable output of the triboelectric sensing unit and capacitive sensing unit.

Then we comprehensively selected and tested 12 samples with diverse shapes, material and Shore hardness levels. Their photographs are illustrated in Fig. S5. Tactile information was extracted from the bi-channel signals to form the dataset for object recognition.

**Fig. 4:**
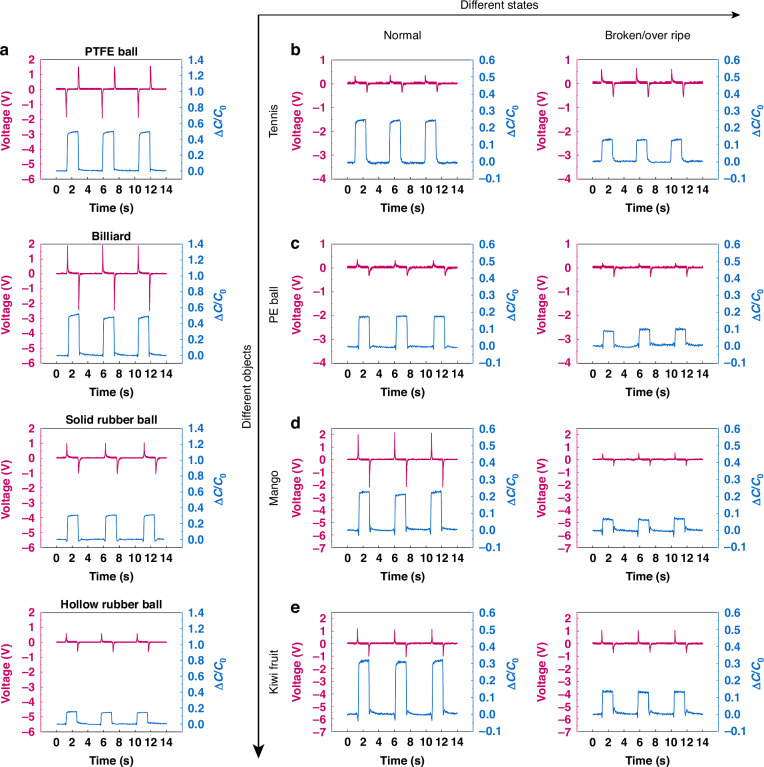
Bi-channel signals of the 12 samples. **a** a PTFE ball, a billiard ball, a hollow rubber ball, and a solid rubber ball, **b** a normal tennis ball and a broken tennis ball, **c** a normal PE ball and a broken PE ball, **d** a normal mango and an overripe mango, **e** a normal kiwi and an overripe kiwi

Figure 4a shows the corresponding bi-channel signals of four kinds of balls, including a PTFE ball, a billiard ball, a hollow rubber ball and a solid rubber ball. Both the PTFE ball and the billiard ball exhibit relatively high Shore hardness, they are difficult to be distinguished solely based on relative capacitance changes. However, they can be easily distinguished since their triboelectric signals have completely opposite polarities. For the hollow and solid rubber balls, significant differences are observed in both the triboelectric and capacitive signals. We also evaluated the different states of four objects, including a tennis ball, a PE ball, a mango, and a kiwi. These objects are susceptible to undergoing changes in their states. They were characterized as a normal state and a broken or overripe state, respectively. The corresponding bi-channel signals of these objects in both states are shown in Fig. 4b–e. In the broken state, both the tennis ball and the PE ball exhibit a reduction in Shore hardness compared to their normal state. Such change is effectively represented by noticeable alterations in capacitance signals from the hybrid tactile sensor. However, their triboelectric signals show less variation. In contrast, significant differences in both triboelectric and capacitance signals can be observed in the overripe mango and kiwi, owing to undergo substantial changes in surface texture and Shore hardness compared to their normal states. This experiment demonstrates that the hybrid tactile sensor is capable not only of discerning the type of objects but also their states.

**Fig. 5:**
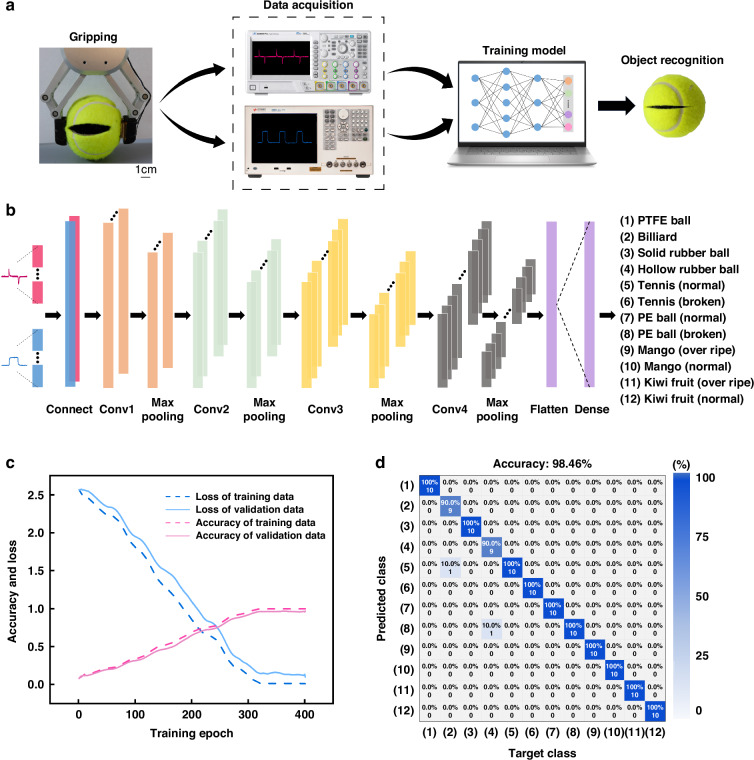
Machine learning-assisted object recognition application with the hybrid tactile sensor. **a** Schematic of the object recognition process. **b** Schematic of the process and parameters for constructing a one-dimensional CNN. **c** Learning curve during the training process. **d** Results of the recognition among the 12 samples shown by prediction confusion matrix

The signals acquired from the tactile sensor have significant potential for object recognition. They contribute to the generation of a valuable training dataset for further analysis and machine learning applications. As a novel data processing and signal analysis methodology that has attracted great attention in recent years, deep learning techniques can greatly enhance recognition capabilities, enabling the identification of a broader range of objects. It builds upon traditional artificial neural networks by increasing model depth and improving data processing and analysis through hierarchical data representations. To improve the recognition accuracy and efficiency, the convolutional neural network (CNN) algorithm was employed to assist the object recognition. The object recognition system we designed is shown in Fig. 5a. We use the bi-channel signals of 12 objects obtained from the above experiments as the dataset. To ensure the accuracy and adequacy of the data, 100 acquisitions were made for each object. These data will be transferred to the computer for deep learning model training.

The structure of the deep learning network we designed is shown in Fig. 5b. We chose 1D-CNN as the feature extractor for our deep learning network. 1D-CNN is highly effective for processing time-series data of the hybrid tactile sensor’s bi-channel signals. These networks capture intricate patterns through convolution operations and enhance the sensor’s recognition capabilities. 1D-CNN enables end-to-end training from raw sensor data to recognition results. It eliminates complex feature engineering and allows automatic learning of relevant patterns. 1D-CNN uses convolutional kernels that slide over the temporal dimension and reduce parameters compared to fully connected networks. This enhances computational efficiency and speeds up the training process. Moreover, 1D-CNN excels at capturing short-term dependencies in time-series data. It is crucial for recognizing subtle and transient changes detected by the tactile sensor. This improves the recognition accuracy by leveraging the complementary nature of both modalities.

We employed a multi-layer 1D-CNN to construct the entire network architecture, which enables the extraction of more complex and abstract features, thereby enhancing the model’s performance. In order to eliminate the effect of scale difference caused by two different magnitude signals during deep learning training, we normalize the friction electric signal and capacitance signal output from the sensor. We splice the normalized friction and capacitance signals into two channels of one-dimensional data as the input to the network.

To avoid the problems caused by sample imbalance, we randomly divide the samples of each object into training and validation sets in the ratio of 9:1. This ensures that the model is able to learn and generalize sufficiently during the training process. As the learning curve shown in Fig. 5c, the training results become stable after 400 training cycles. The accuracy gap between the training and test sets is minimal. This indicates that the network model possesses strong generalization ability and robustness. The confusion matrix of the deep learning network prediction results is shown in Fig. 5d, and the accuracy of the validation set can reach 98.46%. The results demonstrate that the hybrid tactile sensor can accurately accomplish object recognition with the assistance of deep learning. To demonstrate the ability of the proposed hybrid tactile sensor to recognize multiple states of the same object, we added three additional states of tennis balls, which were deflated by 5 ml, 10 ml, and 20 ml, respectively, to simulate the conditions of deflated tennis balls. Along with the normal and broken tennis balls, the 5 states of a tennis ball can be correctly recognized with an accuracy of over 92% (Fig. S6 in supplementary information). Furthermore, to enhance the robustness and generalizability of the deep learning model, 6 new samples were added to the initial dataset, including a raw mango, a rotten mango, a balloon, and three tennis balls in deflated states. The recognition accuracy of the extended set of 18 samples reaches 95.56% (Fig. S7 in supplementary information).

## Conclusion

In this work, we developed a hybrid tactile sensor by integrating a triboelectric sensing unit and a capacitive sensing unit. Both the triboelectric layer and the dielectric layer were prepared by a single porous PDMS process. The hybrid tactile sensor can help the robotic gripper acquire information about the object’s hardness and surface and generate bi-channel signals during gripping. We characterized the sensor’s stability, consistently delivering bi-channel signals under varied stimulation frequencies. Additionally, we explored the optimal mechanical gripping depth of the hybrid tactile sensor on a robotic gripper. We also demonstrated a deep learning-assisted object recognition method that can accurately recognize 18 different samples. By analyzing the training and validation sets, we found that the network performed well on both datasets, showing that the network has good robustness. The deep learning network achieved an accuracy of 98.46% on the validation set. In summary, the hybrid tactile sensor shows good performance in the recognition of objects and provides a reliable solution for achieving accurate recognition.

## Materials and methods

### Preparation of the sensor

Two square acrylic molds with a side length of 15mm and a height of 1 mm and 2 mm, respectively, were first prepared. There are screw holes in the center of the molds to facilitate the fixation of the substrate. Electrodes patterned FCCL was embedded between the two acrylic molds. The base of PDMS (Sylgard 184, Dow Corning) was mixed with NaCl particles in a weight ratio of 1:2, and the curing agent of PDMS was added into the mixture with a weight ratio of 20:1. In order to prepare uniform NaCl particles, NaCl particles of 355–400 μm in diameter were extracted using 0.355 mm and 0.4 mm sieves (GB/T6003.1-2012). Subsequently, the PDMS and NaCl mixture was cast into the acrylic molds and cured in an air oven (PCHB-C6000 Serials) at 80°C for 120 minutes. The molds were then removed after the PDMS films were cured. The PDMS films were then bathed in water at 50°C in an ultrasonic cleaner (Fisherbrand, 11205) to obtain porous PDMS. Finally, the FCCL substrate was folded and pasted, and the hybrid tactile sensor was fabricated.

### Characterization setup

The mechanical excitations were applied to the sensor by a dynamic test instrument (Instron, E1000). In order to maintain the same gripping depth on different objects, the route and position of the gripper were pre-programmed to match the size differences of these objects. The voltages generated by the triboelectric sensing unit were measured by a digital oscilloscope (ZLG, ZDS2024B) via a 100:1 oscilloscope probe. A low-noise current amplifier (SRS, SR570) is used to measure the currents of the triboelectric sensing unit. The capacitance of the capacitive sensing unit is measured by an impedance analyzer (Keysight, E4990A). In addition, the surface topography of the porous PDMS was observed using a scanning electron microscope (Hitachi, SU8010).

## Supplementary information

Supplementary information

supporting video1

supporting video2
