# Supplementary material for: Deep learning-assisted object recognition with hybrid triboelectric-capacitive tactile sensor
Source: Microsyst Nanoeng. 2024 Nov 7;10:165. doi: 10.1038/s41378-024-00813-2 (PMC11543809; doi:10.1038/s41378-024-00813-2)
Supplement: Supplementary file 1 — Supplementary information [file 41378_2024_813_MOESM1_ESM.docx]

**Supplementary information**

**Deep Learning-Assisted Object Recognition with Hybrid Triboelectric-Capacitive Tactile Sensor**

Yating Xie^1,2^, Hongyu cheng^1,2^, Chaocheng Yuan^1^, Limin Zheng^1^, Zhengchun Peng^1^, Bo Meng^1,*^

1 Key Laboratory of Optoelectronic Devices and Systems of Ministry of Education and Guangdong Province, College of Physics and Optoelectronic Engineering, Shenzhen University, Shenzhen 518060, China

2 These authors contributed equally: Yating Xie, Hongyu cheng

* Corresponding author: [bomeng@szu.edu.cn](mailto:bomeng@szu.edu.cn)

# Supporting Figures


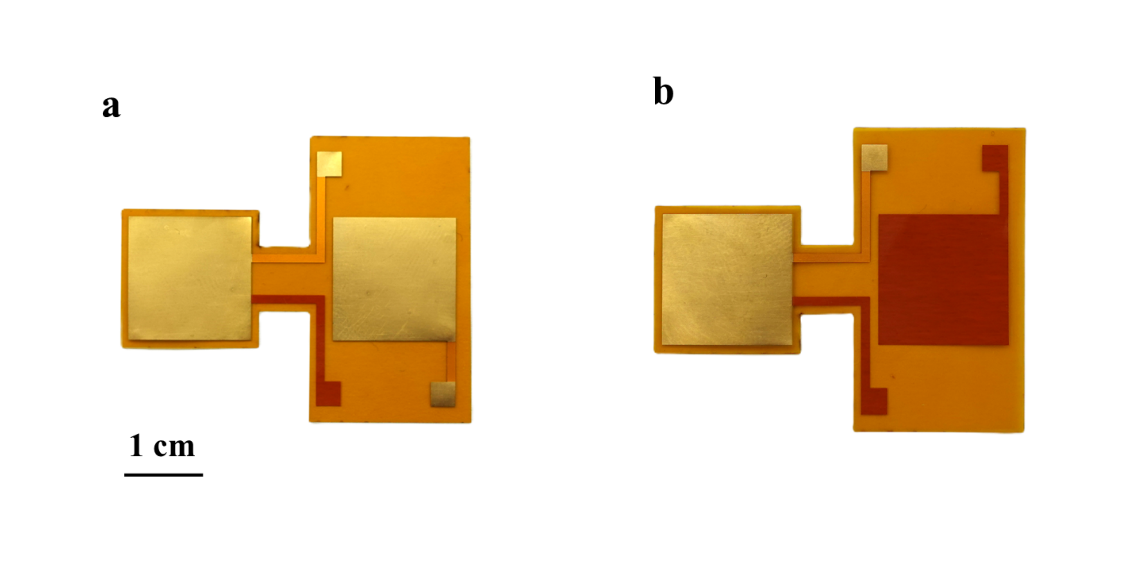


**Figure S1 a** Structural and **b** dimensional details of the hybrid tactile sensor.


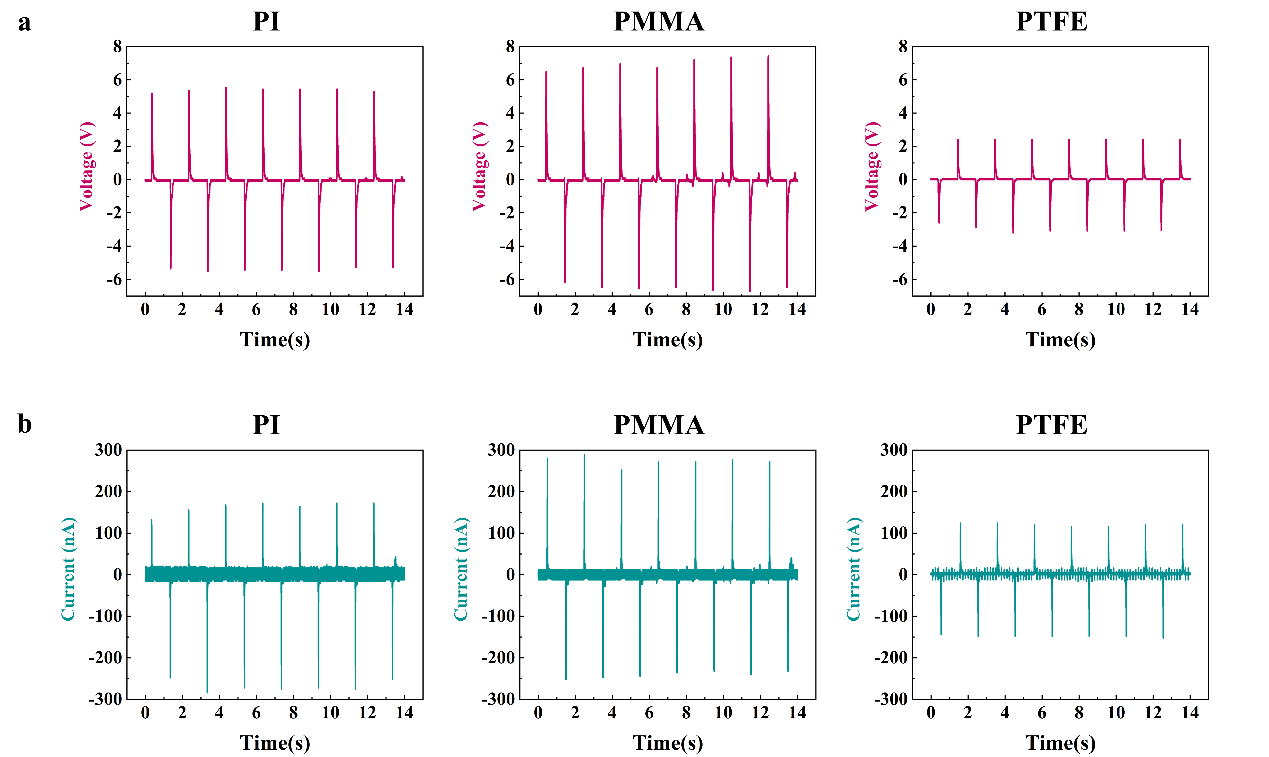


**Figure S2** Characterizations of the tactile sensor in contact with different materials. **a** Voltage and **b** short-circuit current outputs of the sensor under 0.5 Hz mechanical stimulus.


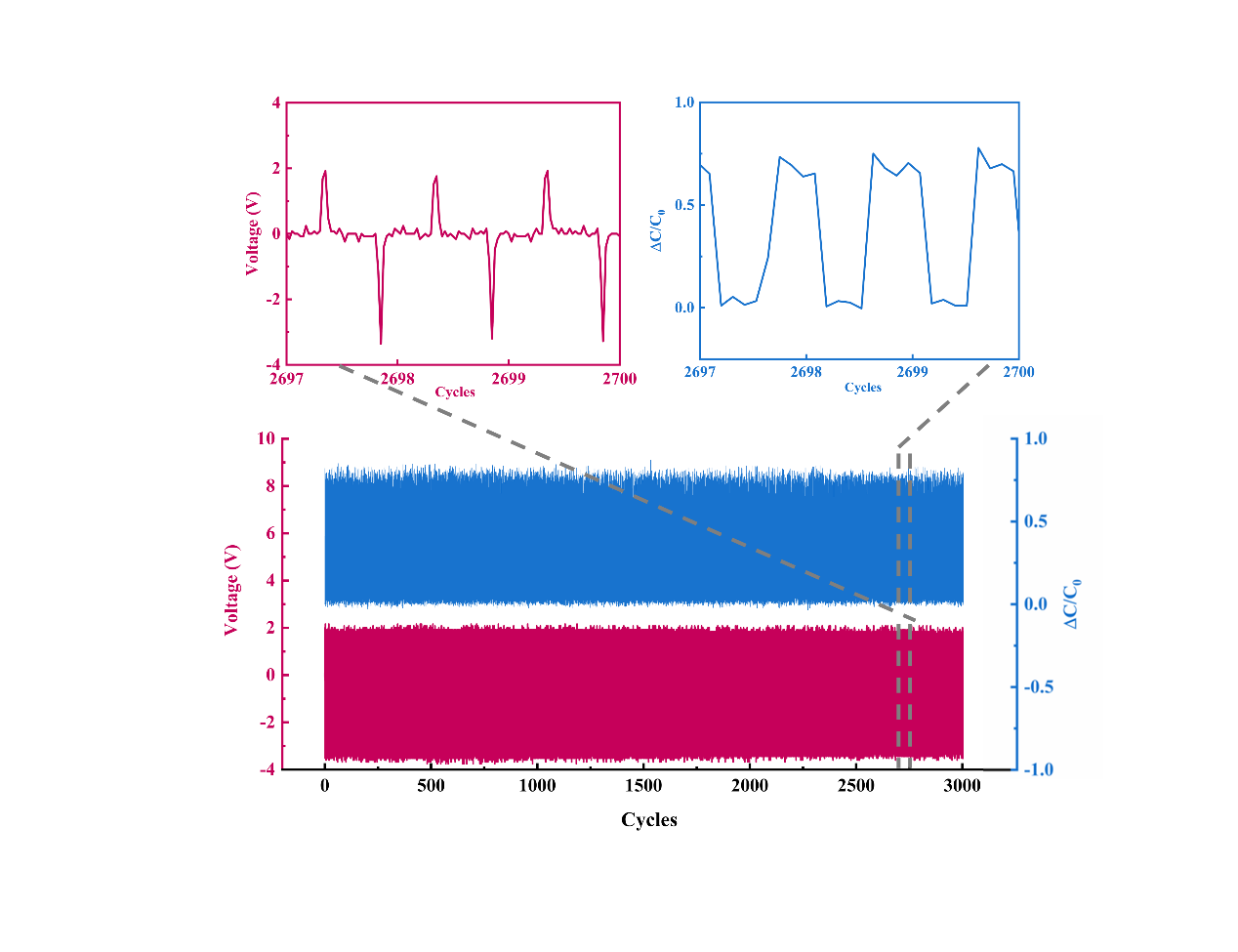


**Figure S3** Bi-channel output of triboelectric and capacitive signals under 3000 repeated cycles at a frequency of 0.5 Hz.


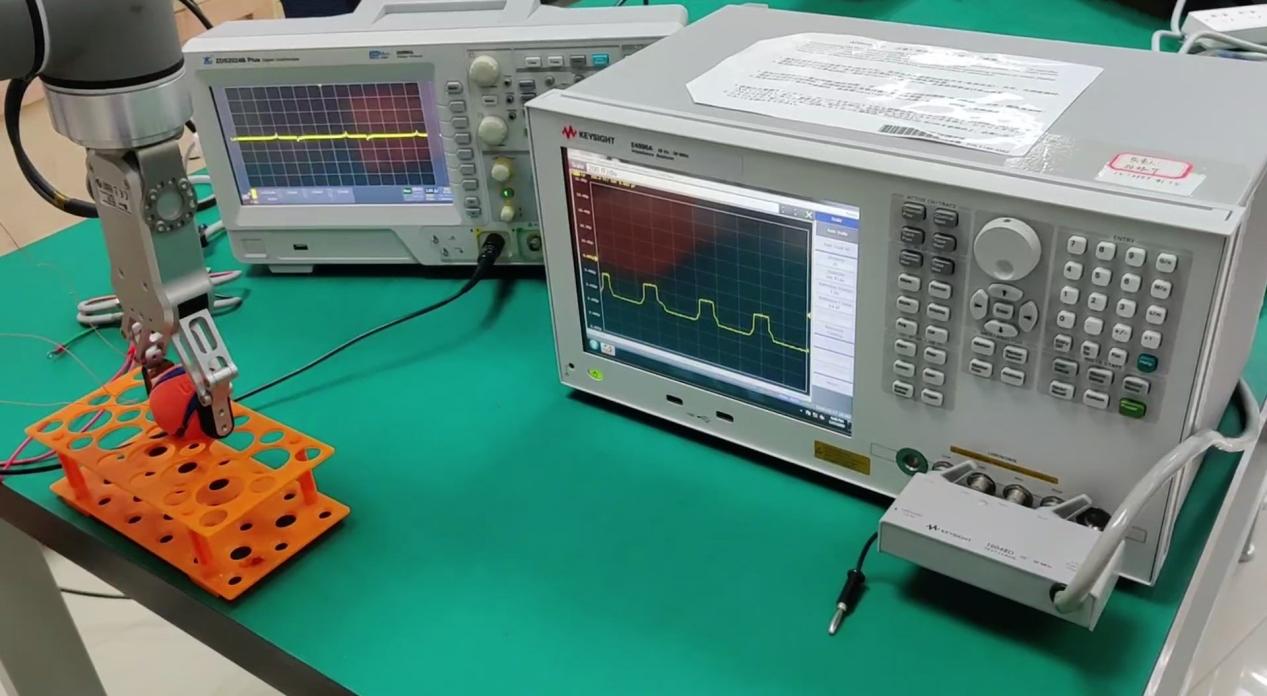


**Figure S4** Setup of the tactile sensor’s bi-channel signal acquisition.


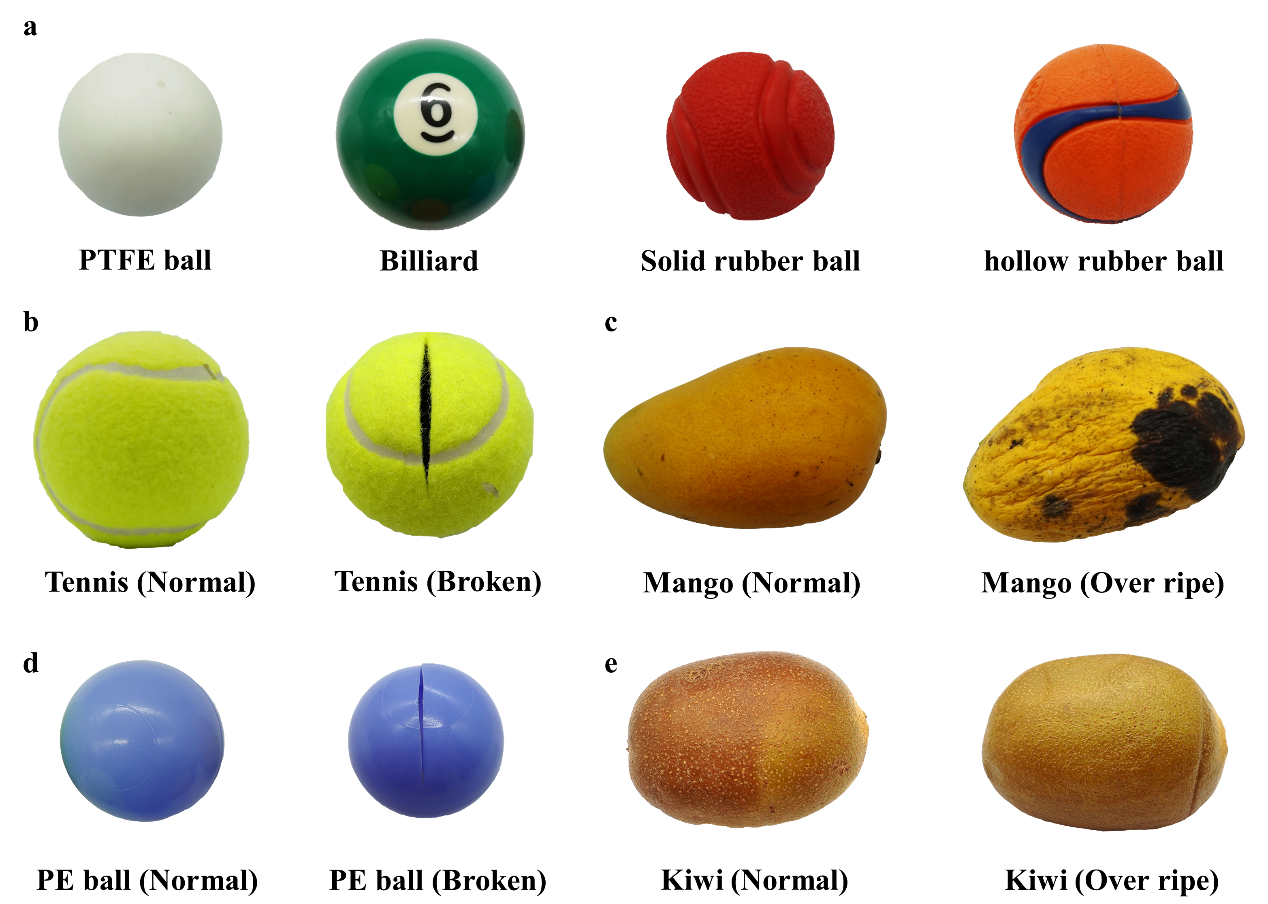


**Figure S5** Photographs of 12 samples used in object recognition experiments. Four kinds of different balls. **b** A normal and a broken tennis ball. **c** A normal and an over ripe mango. **d** A normal and a broken PE balls. **e** A normal and an over ripe kiwi.


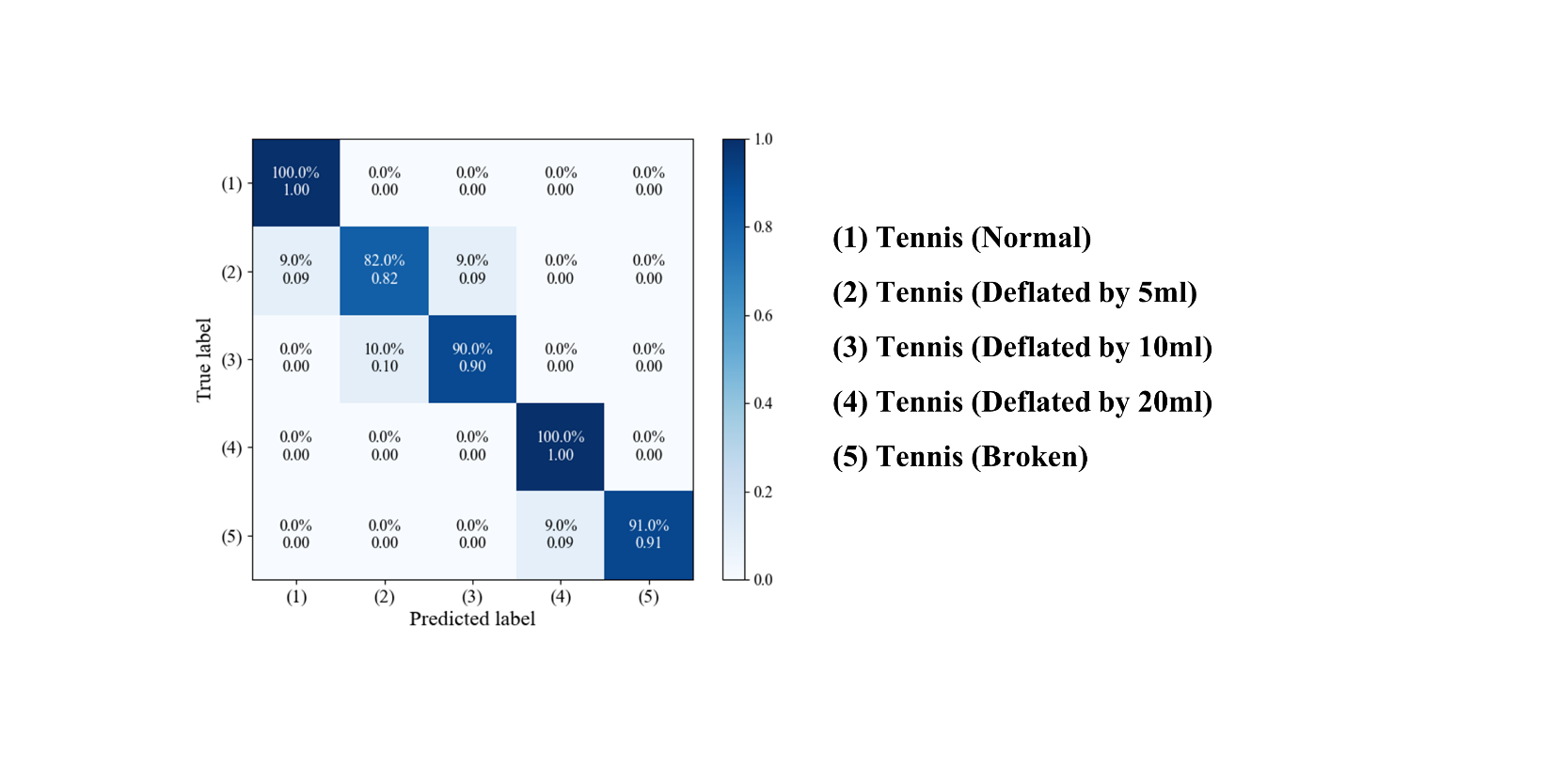


**Figure S6** Prediction confusion matrix of the recognition results among five tennis samples in different states.


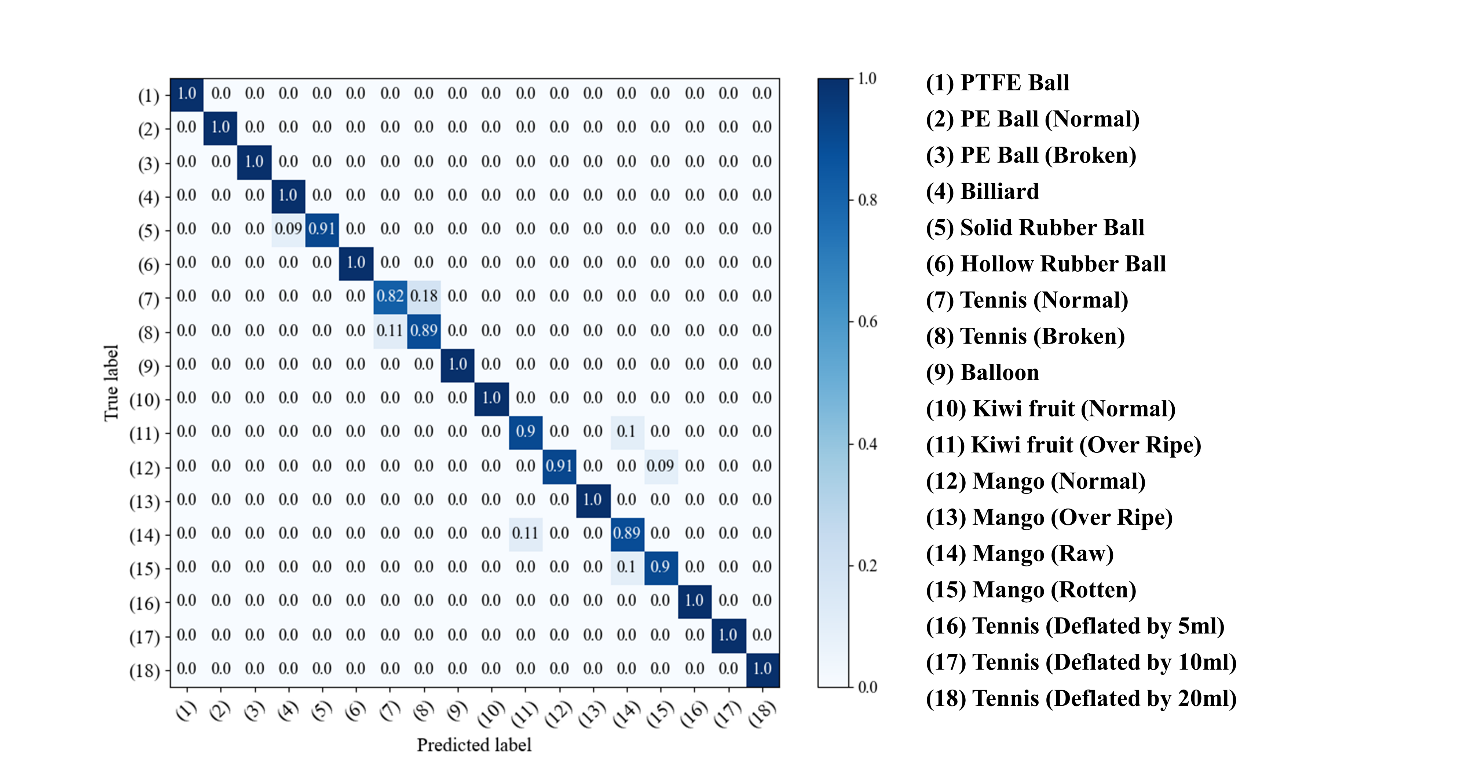


**Figure S7** Prediction confusion matrix of the recognition results among an extended set of 18 samples.
